# Evaluating the Components, Nutrients, and Antioxidant and Anti-Inflammatory Properties of *Centranthera grandiflora* Benth Extracts

**DOI:** 10.3390/nu17050925

**Published:** 2025-03-06

**Authors:** Wenjuan Yuan, Xinlan Liu, Xinting Wang, Zejin Nian, Xiaoyun Wu, Chengting Zi, Sha Xu, Xiaojing Shen, Xuanjun Wang

**Affiliations:** 1 College of Science, Yunnan Agricultural University, Kunming 650201, China; yuanwj0805@126.com (W.Y.); nq2405542406@163.com (Z.N.); wuxiaoyun79@163.com (X.W.); 2 Key Laboratory of Puer Tea Science, Ministry of Education, Yunnan Agricultural University, Kunming 650201, China; liuxinlan15@163.com (X.L.); 18787628093@163.com (X.W.); zichengting@126.com (C.Z.); 3 College of Food Science and Technology, Yunnan Agricultural University, Kunming 650201, China; 4 College of Resources, Environment, and Chemistry, Chuxiong Normal University, Chuxiong 675000, China; 19887436424@163.com

**Keywords:** *Centranthera grandiflora* Benth, antioxidant activity, anti-inflammatory activity, chemical constituents, nutritional value, molecular mechanisms

## Abstract

Background: *Centranthera grandiflora* Benth is commonly utilized in China to take advantage of its purported health benefits. Methods: Here, the chemical composition, nutritional value, and bioactivity of *C. grandiflora* Benth extract (CGE) are characterized, and the mechanisms through which it functions were explored. Results: CGE was found to exhibit a favorable nutritional and biosafety profile, especially due to its high amino acid and mineral contents. A UPLC-ESI-Q-TOF/MS approach identified 20 compounds. Through network pharmacology analyses, the antioxidant activity of CGE was found to be mediated through the PI3K/Akt pathway, with molecular docking results providing support for mussaenoside and azafrin as important bioactive compounds. At the cellular level, antioxidant activity of key protective antioxidants including GSH-Px and SOD while suppressing ROS accumulation, levels of damage-related factors (MDA, NO, TNF-α, IL-1β, and IL-6), and iNOS and COX-2 in RAW264.7 cells treated with LPS. These findings offer potential evidence for using CGE to lower oxidative stress and inflammation. Further analyses demonstrated the ability of CGE to promote Nrf2 and HO-1 upregulation, whereas Keap1 levels were suppressed, as were PI3K/Akt/NF-κB proteins. In light of these results, CGE appears to be able to act via simultaneously enhancing Nrf2/HO-1 activity and reducing that of PI3K/Akt/NF-κB. Conclusions: CGE, as a rich source of iridoid glycosides and other nutrients, may thus be a valuable dietary supplement for use in food applications.

## 1. Introduction

There has been growing public, research, and industry interest in health-enhancing products in recent years [1]. The genus *Centranthera* (family: Scrophulariaceae) includes seven species distributed from India through Malaysia and Oceania, of which four are reportedly present in China [2]. *Centranthera* species have been used as both food and medicinal sources for improving overall health and longevity in traditional Chinese medicine (TCM) practices for several thousand years, as evidenced by their incorporation in established crude medicinal preparations such as *Rehmannia glutinosa* and Scrophulariaceae present in the Chinese Pharmacopoeia (2020 edition) [3]. *Centranthera grandiflora* Benth, the “wild bean root”, was first described in “Chinese Materia Medica”. The roots of the plant are commonly used in folk medicine practices in Yunnan, Guizhou, and Guanxi to alleviate pain, reduce swelling, arrest bleeding, and disperse blood stasis [4,5]. Given these promising properties, there is a clear opportunity to develop novel drugs and health foods based on *C. grandiflora* Benth for hepatoprotection and the treatment of conditions including cardiovascular diseases, leukemia, diuresis, hypotension, and hyperglycemia [6].

Recent clinical and pharmacological research efforts have provided evidence in support a variety of biological properties attributable to *C. grandiflora* Benth, including the ability to prevent myocardial ischemia [7], together with hepatoprotective [8], anti-inflammatory [9], antioxidant [10,11], and other medicinal activities. The main bioactive compounds present in *C. grandiflora* Benth include iridoid glycosides, carotenoids, phenylethanol glycosides, ionone glycosides, and monoterpenoid glycosides [4,10,12]. Of these, iridoid glycosides are the most abundant, consistent with the chemical characteristics of plants in the Scrophulariaceae family. These glycosides reportedly exhibit anticancer, hypoglycemic, and anti-inflammatory activity. *C. grandiflora* Benth has been evaluated as a natural medicine and has been shown to be effective as an antioxidant and against inflammation [13,14]. Some of the earliest studies of this plant revealed its ability to alleviate alcohol-induced hepatic injury in mice through increases in antioxidant enzyme levels and the resultant mitigation of oxidative stress [10]. Azafrin, which is a key component present in *C. grandiflora* Benth, is capable of attenuating H_2_O_2_-induced oxidative injury through increases in antioxidant protein expression, ultimately protecting against ischemic heart disease in experimental mice [7]. *C. grandiflora* Benth can also reportedly significantly reduce intracapsular inflammatory PGE_2_ levels in a model of carrageenan gum-induced air sac synovitis in mice, consistent with robust anti-inflammatory activity [9].

Excessive ROS production in biological systems can trigger oxidative stress, resulting in consequent inflammation. Elevated levels of ROS biogenesis can also induce the upregulation of SOD, MDA, and GSH-Px [15]. Heme oxygenase (HO)-1 can protect against oxidative stress resulting from ROS accumulation [16]. The Keap1/Nrf2 axis increases antioxidant enzyme levels, leading to HO-1 upregulation and reduced sensitivity to inflammatory damage caused by oxidative stress [17,18]. NF-κB activation can also trigger NO and inflammatory mediator production, including the upregulation of major inflammatory proteins including COX-2 and iNOS. Suppressing NF-κB signaling thus holds promise as an approach to mitigating inflammation [19].

*C. grandiflora* Benth has been the focus of growing interest as a food and medicinal herb, although its components and their effects are not well-understood. To promote the further utilization of this plant, the present study employed a UPLC-ESI-Q-TOF-MS approach to characterize the phytochemical makeup and nutritional value of *C. grandiflora* Benth extract (CGE) samples. The safety of this extract was assessed through analyses of acute toxicity and cytotoxicity, while measurements of its properties together with the underlying mechanisms of action were conducted through network pharmacology, Western blotting, and other analyses. Together, these results may emphasize the utility of *C. grandiflora* Benth, providing a basis for its sustainable development as a valuable natural resource.

## 2. Materials and Methods

### 2.1. Reagents

*C. grandiflora* Benth were harvested from Heping Township, Pingbian County, Yunnan Province in July 2023, after which it was identified by Dr. Gao Yanmei of the Honghe Prefecture Inspection and Testing Institute (Yunnan Province) and deposited in the Key Laboratory of the Ministry of Education of Pu’er Tea Science of Yunnan Agricultural University. All chemicals used for this study were obtained from Tianjin Damao Chemical Reagent Factory (Tianjin, China).

RAW264.7 cells were from the Shanghai Cell Bank of the Chinese Academy of Sciences. DPPH, ABTS, dexamethasone (DXMS) and Nuclear Protein Extraction Kit (100T) were from Solarbio Bioscience & Technology Co., Ltd. (Shanghai, China). DMEM, penicillin-streptomycin, and FBS were from Hyclone (Thermo Fisher, Waltham, MA, USA). LPS (*Escherichia coli* O55:B5) was from MedChemExpress (Monmouth Junction, NJ, USA). ELISA kits for IL-6, TNF-α, and IL-1β and all utilized antibodies (Keap1, Nrf2, HO-1, iNOS, COX-2, p-Akt, Akt, p65, p-p65, IκBα, p-IκBα, LaminB) were from ABclonal, Technology (Beijing, China), while assay kits for NO, SOD, MDA, and GSH-Px were from Abbkine Scientific Co., Ltd. (Wuhan, China).

MS-HRESI was conducted with an Agilent 1290/6540 UPLC/Q-TOF-MS instrument, while a used for MS-HRESI, while an Agilent Technologies 1200 Series chromatography system (Agilent, Santa Clara, CA, USA) coupled with a UV-Vis detector and an Agilent ZORBAX SB-C18 column (250 × 4.6 mm, 5 µm) column was employed for semi-preparative HPLC. Column chromatography (CC) was conducted with Sephadex LH-20 (40–70 μm, Cytiva, Marlborough, MA, USA), RP-18 (LiChroprep, 100 Å, 40–63 μm, Merck, Union County, NJ, USA), and silica gel (100–200 and 300–400 mesh, Qingdao Marine Chemical, Inc., Qingdao, China).

### 2.2. Crude C. Grandiflora Benth Extract (CGE) Preparation

The dried roots (1.5 kg) of *C. grandiflora* Benth. were crushed and extracted with 65% ethanol by heating samples for 4 h at 60 °C (3 × 15 L). The resultant was condensed under low pressures, yielding the dried ethanolic extract (668.18 g) and lyophilized for subsequent experiments.

### 2.3. Characterization of Phytochemical Composition

#### 2.3.1. UPLC-ESI-Q-TOF/MS

Samples were prepared by weighing an appropriate amount of CGE and suspending in methanol solution and diluted to 200 µg/mL. An instrument (1290 Infinity II, Agilent, Santa Clara, CA, USA) with a quadrupole time-of-flight mass analyzer (QTOF, Agilent, Santa Clara, CA, USA) and a ZORBAX SB-C18 RRHD column (250 × 4.6 mm, 5 µm, Agilent, Santa Clara, CA, USA) was utilized for these analyses. Elution involved a gradient of 0.1% formic acid (A) and acetonitrile (B) with settings of 0–45 min, 2–100% B; 46–50 min, 100% B, with a 1.0 mL·min^−1^ flow rate. The temperature was maintained at 40 °C, with a total run time of 50 min. An Agilent 6540 Q-TOF instrument with a dual electrospray ionization source (ESI) operated in negative and positive ion modes was used to acquire mass data. For these analyses, the scan time was 2 spectra pers, the desolvation gas rate was 10 L·min^−1^ at 350 °C, and the nebulizer pressure was 40 psi. The respective fragment and capillary voltages were 135 V and 3.5 kV. The Mass Hunter Qualitative Analysis Program B.08.00 (Agilent) was utilized to analyze raw mass spectra.

#### 2.3.2. Proximate Composition Analyses

A previously reported approach was used to assess the proximate composition of CGE [20,21]. Briefly, after extracting crude lipids, they were analyzed with a Hanon SOX606 Automatic Soxhlet extraction analyzer (Hanon, Changchun, China), while measures of nitrogen content were made with the Prosky-AOAC method and a Kjeltec 8400 nitrogen analyzer (FOSS, Hillerød, Denmark), using this approach to compute total protein concentrations. National food safety standards (GB 5009.88-2023 [22]) were used to guide the measurement of total dietary fiber. Analyses included three replicates. Digestible carbohydrate levels were computed with Formula (1):

Digestible carbohydrate content = 100 − (CL% + CP% + DB% + AS%)(1)

where CL denotes the amount of crude lipid, CP the amount of crude protein, DB the amount of dietary fiber, and AS the amount of ash.

#### 2.3.3. Mineral Analysis

An AOAC method described previously was used to assess CGE mineral composition [23]. Briefly, HCl and water were used to digest dried *C. grandiflora* Benth. in a closed-vessel microwave digestion system. The resultant samples were filtered, placed in a volumetric flask, and water was added to an appropriate volume, followed by the use of inductively coupled plasma mass spectrometry (ICP-MS) (NexION 300D, PerkinElmer, Shelton, CT, USA) for the identification of selenium (Se, 196.0 nm), phosphorus (P, 213.6 nm), zinc (Zn, 213.8 nm), iron (Fe, 248.3 nm), manganese (Mn, 279.5 nm), magnesium (Mg, 285.2 nm), copper (Cu, 324.8 nm), calcium (Ca, 422.6 nm), sodium (Na, 589.6 nm), and potassium (K. 766.5 nm) in these samples. Standard curves were employed to quantify the amounts of these minerals in the analytes. Analyses were performed with three replicate samples, reporting the amounts of minerals as mg/100 g sample.

#### 2.3.4. Analyses of Amino Acid Composition

After adding CGE to a 20 mL ampoule containing 10 mL of 6 M HCl, samples were snap-frozen with liquid nitrogen, followed by the sealing of the ampoule and its transfer into an oven to undergo hydrolysis for 24 h at 110 °C. Following the subsequent filtering of samples, supernatants were transferred into a bottle and evaporated until dry under vacuum at 60 °C in an evaporimeter. Samples were next dissolved with 5 mL of sodium citrate buffer (pH 2.2; *c*Na^+^: 0.2 mol·L^−1^) and centrifuged, followed by analysis of the supernatants on a Biochrom 30+ Automatic Amino Acid Analyzer (Biochrom, Cambridge, UK), detecting the amino acid composition using an ion-exchange column with a detection wavelength of 570 nm for amino acids apart from proline (440 nm). Standard amino acid solutions (Sigma, St. Louis, MO, USA) were prepared at 1.25 μmol·mL^−1^ in sodium citrate buffer [24].

### 2.4. Acute Toxicity Analyses

The up-and-down method was used to assess the acute toxicity of CGE, with approval from the Institute of Animal Care and Use Committee of Yunnan Agricultural University (approval code: 202407005, dated 1 July 2018). For these analyses, Kunming mice (18–22 g, 10 male, 10 female) from the Laboratory Animal Center of Yunnan University were randomly assigned to 2 groups according to sex and body weight, and acclimatised for 7 days in a SPF grade animal laboratory, with the mice being kept ad libitum with food and water throughout the study period. To treat these animals, CGE was crushed to 200 mesh size and dissolved in distilled water, after which it was administered orally to these mice at 2000 mg·kg^−1^, while control mice were administered an equal volume of saline. Behavioral changes were monitored continuously within 4 h after dosing, and mortality was monitored for 24 h after dosing [25]. Experimental procedures and handling were performed according to the animals’ guidelines [26].

### 2.5. Network Pharmacology Analyses

The results of phytochemical composition analyses led to the selection of 20 compounds as potential drug targets, obtaining information on targets from PubChem and utilizing STRING for protein–protein interaction (PPI) network development. Functional analysis of target genes was conducted with the DAVID database, while Cytoscape 3.8.2 was used for target pathway visualization.

### 2.6. Molecular Docking

Molecular docking for target proteins of interest and binding energy calculations were conducted with AutoDock 1.5.7 and Discovery Studio 2019 client. Binding capacity was assessed based on affinity (kcal·mol^−1^) values, followed by Pymol 2.5 visualization.

### 2.7. Assessment of Antioxidant Activities

CGE samples were prepared by weighing an appropriate amount of CGE and suspending in a solution and stepwise dilution to 320, 160, 80, 40, 20, and 10 μg/mL for DPPH and ABTS experiments, and 800, 400, 200, and 100 μg/mL for FRAP assay.

#### 2.7.1. DPPH Scavenging Assay

DPPH assays were performed as described with slight changes [27]. Briefly, a 100 μL volume of sample solutions of varying concentrations were combined in 96-well plates with DPPH working solution (100 μL; 0.3 mmol/L), followed by incubation at 37 °C for 30 min away from light. Absorbances at 517 nm were read in a microplate reader, utilizing ethanol (negative control, 100 μL of anhydrous ethanol and 100 μL of DPPH working solution) and vitamin C (Vc, positive control, 100 μL of Vc solution and 100 μL of DPPH working solution). The scavenging activity of DPPH was then calculated using Equation (2):

(2) $$R=\left(1-\frac{A_{1}-A_{2}}{A_{d}}\right)\times 100\%$$

where *R* denotes scavenging activity; *A*_1_ is the absorbance for the combination of the sample and the DPPH solutions; *A*_2_ represents the absorbance of the sample solution mixed with equivalent amounts of anhydrous ethanol; and *A*_d_ represents the absorbance of a mix of DPPH and anhydrous ethanol (1:1).

#### 2.7.2. ABTS Assays

ABTS assays were performed as described [27] with minor changes. Specifically, 100 µL of ABTS solutions of varying concentrations were mixed with equal volumes of samples (30 min, 37 °C, away from light) before reading absorbances at 734 nm. In addition, ethanol was utilized as a negative control (100 μL of anhydrous ethanol and 100 μL of ABTS working solution) and VC was utilized as a positive control (100 μL of Vc solution and 100 μL of ABTS working solution). ABTS scavenging activity was then calculated as in Equation (3).

(3) $$R=\left(1-\frac{A_{3}-A_{4}}{A_{a}}\right)\times 100\%$$

where *R* denotes scavenging activity; *A*_3_ corresponds to absorbance for the combined sample and ABTS solutions; *A*_4_ represents the absorbance of the sample solution mixed with equal amounts of anhydrous ethanol; and *A*_a_ represents the absorbance of ABTS working solution together with equal volumes of anhydrous ethanol.

#### 2.7.3. FRAP Assay

FRAP assays were performed as described [28] with some modifications. Briefly, a working solution of FRAP (150 µL, composed of 300 mmol/L acetate buffer (pH 3.6), 10 mmol/L TPTZ solution (prepared with 40 mmol/L HCl, 20 mmol/L FeCl_3_ solution in the ratio of 10:1:1 by volume) was combined for 30 min with 5 µL of sample solutions of varying concentrations at 37 °C and absorbances at 593 nm were read. Then, FeSO_4_ standard curves were generated over 0.1–1.0 mmol/L, allowing for the measurement of FRAP activity in FeSO_4_ equivalents mmol/L.

### 2.8. Cellular Analysis of the Antioxidant and Anti-Inflammatory Properties of CGE

#### 2.8.1. Cells

RAW264.7 cells were grown in DMEM with 10% FBS and 1% penicillin/streptomycin at 37 °C with 5% CO_2_.

#### 2.8.2. Cell Viability

An MTT assay protocol reported previously was employed to assess the viability of macrophages treated using CGE [29]. Briefly, after inoculation of RAW264.7 cells (2 × 10^5^ well) in 96-well plates, these cells were grown for 12 h, before inclusion of CGE (3.25–400 µg/mL) for 24 h. Then, 20 μL/well of MTT solution was introduced, and following a 4 h incubation and replacement of the media with DMSO (200 μL), absorbances at 492 nm were read.

#### 2.8.3. Intracellular ROS Analyses

DCFH-DA staining was used for analyses of ROS production [30]. Briefly, after pretreating RAW264.7 cells for 2 h with various sample solution concentrations, these cells were dosed using LPS (1 µg/mL) for 18 h, followed by DCFH-DA (1 mM) for 30 min at 37 °C, after which fluorescence microscopy was used to assess ROS levels.

#### 2.8.4. MDA, SOD, and GSH-Px Analyses

MDA, SOD, and GSH-Px are key indices for antioxidant activity [31]. Their levels were determined with kits as directed by the manufacturer.

#### 2.8.5. Analyses of NO and Inflammatory Cytokine Production

Levels of NO and inflammatory cytokine levels were measured with slightly modified versions of prior protocols [32]. Briefly, RAW264.7 cells were grown overnight in 6-well plates, followed by sample solution and LPS treatment as above. NO levels in cell supernatants were then detected with the Griess reagent, while ELISAs were used for the detection of TNF-α, IL-6, and IL-1β.

#### 2.8.6. Western Immunoblotting

RAW264.7 cells were grown for 2 h with varying amounts of samples before stimulation with 1 µg/mL LPS for 18 h. After rinsing with PBS, RIPA buffer with PMSF (1:100) was used to extract the proteins therein for 30 min. A BCA kit (Biotopped, Beijing, China) was then employed for protein quantification, and proteins were separated through 10% SDS-PAGE before transfer to PVDF membranes which were blocked (5% non-fat milk, 1 h, room temperature) and treated with primary antibodies (Keap1, Nrf2, HO-1, iNOS, COX-2, p-Akt, Akt, p65, p-p65, IκBα, p-IκBα, and LaminB) at 4 °C. Membranes were then rinsed using TBST three times, treated with HRP-conjugated secondary antibodies (2 h, room temperature), visualized with the BeyoECL Plus luminescent reagent (Biosharp, Beijing, China), and analyzed with ImageJ (NIH, Bethesda, MD, USA).

### 2.9. Statistical Analysis

Data from triplicate experiments are shown as means ± SD. GraphPad Prism 5.0 and SPASS 24.0 (Origin Institute Inc., Grand Prairie, TX, USA) were used for statistical comparisons, with *p* < 0.05 being used to define significance.

## 3. Results and Discussion

### 3.1. Phytochemical Analysis

#### 3.1.1. UPLC-ESI-Q-TOF/MS Analysis

**Figure 1:**
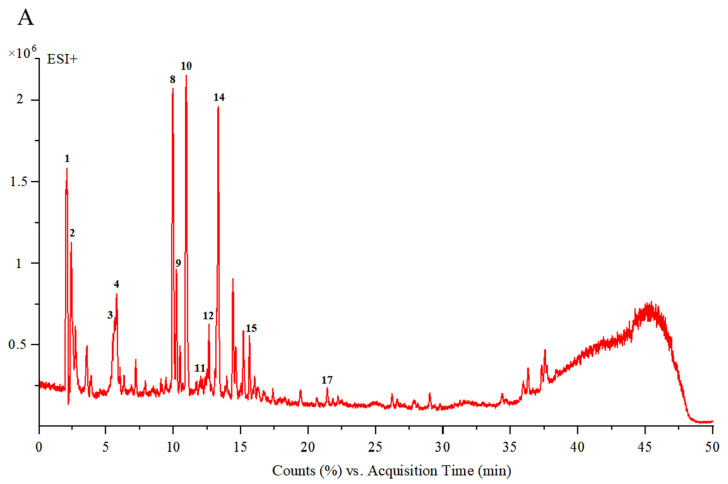

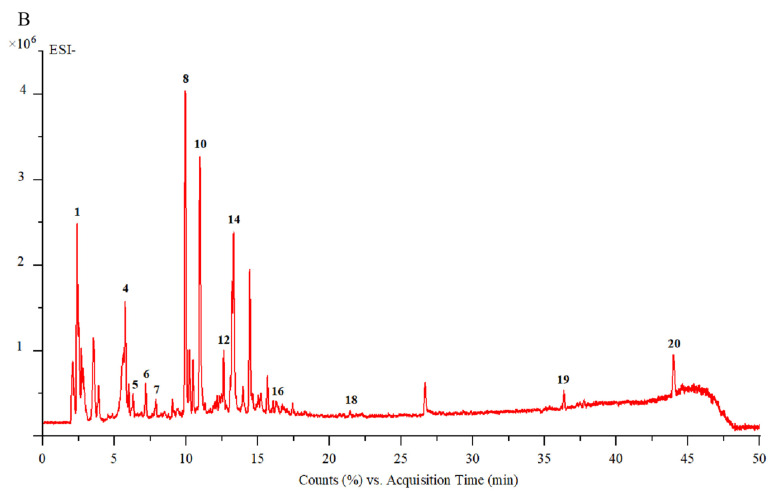
UPLC-ESI-Q-TOF/MS chromatograms of CGE. Positive (**A**) and negative (**B**) ion modes. Numbers 1–20 represent different chemical compositions.

UPLC-DAD-ESI-MS/MS was used to assess the chemical composition of CGE. LC/MS chromatographic peaks for positive and negative ions are shown in Figure 1A,B, while data corresponding to the times of retention, ions, MS2 fragments, compound annotations, and molecular structures are presented in Table 1. Comparisons of the mass spectra data and published datasets led to the identification of 20 compounds, which included iridoid glycosides, amino acids, monoterpenoid glycosides, triterpenoids, flavonoids, alkaloids, disaccharides, and diols.

As shown in Table 1, CGE samples included three amino acids (glutamate, aspartic acid, and leucine) that were also detected in tests of nutritional value. CGE was also found to contain one alkaloid (vomilenine), one triterpenoid (ursolic acid), one diol (mannitol), and one disaccharide (2-methoxy-4-methylphenyl-O-*β*-D-apiofuranosyl-(1→6)-*β*-D-glucopyranoside). It additionally contained three flavonoids (Vitexin 2″-O-rhamnoside, rutin and sophoricoside), monoterpenoid glycosides (centrantheroside A, melasmoside, azafrin, trihydroxy-*β*-violetone, dihydroxy-*β*-violetone), and other iridoid glycosides, which included aucubin, geniposidic acid, mussaenosidic acid, mussaenoside, and catalpol. These monoterpenoid and iridoid glycosides have the potential to provide anti-inflammatory and antioxidant activity (Table 1).

#### 3.1.2. Proximate Compositional Analyses

The CGE proximate compositional analysis findings are illustrated in Table 2. Of the detected components, carbohydrates were most abundant (43.00%), followed by dietary fiber (35.94%), ash (12.26%), protein (8.77%), and moisture (8.67%), whereas lipid levels were extremely low (0.04%). These results align with the frequent consumption of CGE and its extraction using white wine by village elders for use in cardiovascular disease treatment and other healthcare applications. Delays in harvesting time may result in a rapid increase in the levels of dietary fiber in CGE, suggesting a need to more closely explore this link between CGE proximate composition and harvest season.

#### 3.1.3. Mineral Composition Analyses

As demonstrated in Table 2, CGE samples contained a total mineral content of 2601.03 mg per 100 g. The most abundant of these minerals was K (760.02 mg per 100 g), with these levels falling below the recommended daily intake (1800–2000 mg·day^−1^). Mg was the second most abundant mineral (443.14 mg per 100 g), with these levels being above the recommended daily intake (200–330 mg·day^−1^). The respective levels of Fe and Ca were 292.72 and 276.76 mg/100 g. These and other minerals are important for the preservation of physiological functions including bone and tooth health, cardiovascular function, and immune homeostasis.

#### 3.1.4. Amino Acid Composition Analyses

Amino acids comprise proteins, with protein quality being shaped by the amino acid profile such that good quality protein is vital for dietary balance as a source of appropriate nutrients. Here, CGE was found to contain 14 amino acids (total content level: 2.89%). Essential amino acids can only be obtained through dietary intake and are required for human health. Apart from tryptophan and methionine, six essential amino acids were found at levels of 1.12%. CGE was also a rich source of hydrophobic amino acids (1.13%), which exhibit antioxidant activity and can limit disease risk. These findings demonstrate the ability of CGE as a source of amino acids and nutrients, thereby serving as a dietary supplement that can enhance immune function to protect the body.

#### 3.1.5. Toxicity

The safety profile of CGE was evaluated through acute toxicity studies performed with Kunming mice. No abnormalities (decreased appetite, agitation, convulsions, decreased mobility, paralysis) or deaths were observed in mice orally dosed with CGE at 2000 mg/kg over the continuous monitoring time range (4,8,12,16,20,24 h), and the LD50 was thus determined to be greater than 2000 mg/kg. At the dose used in this study, CGE was thus determined to be safe.

### 3.2. Network Pharmacology

**Figure 2:**
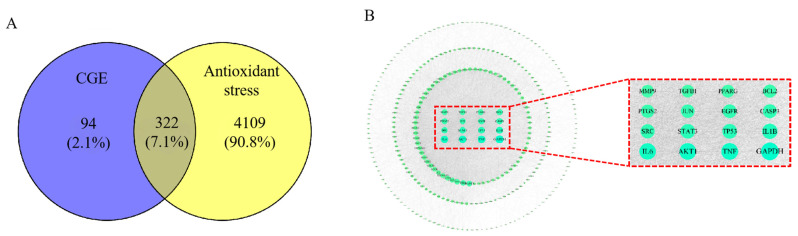

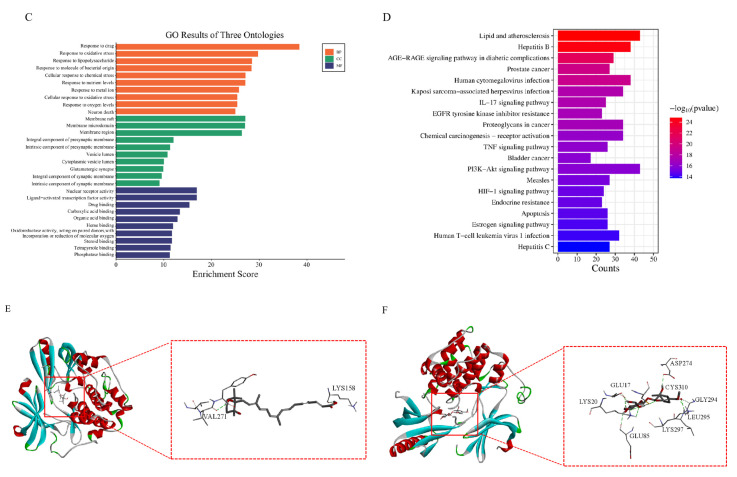
The cyberpharmacology studies and molecular docking tests of CGE. Intersection of components and disease targets (**A**). PPI networks of core targets (**B**). GO enrichment of core targets (**C**) and KEGG pathway analyses (**D**). Molecular docking tests of core compounds azafrin (**E**) and mussaenoside (**F**) with AKT.

The possible mechanisms through which CGE may function were explored through network pharmacology analyses. Potential targets were identified by using the 20 identified compounds as candidates and searching the SWISS TARGET database, yielding 416 targets acting with these compounds. Additionally, 4431 targets related to antioxidant stress were identified in the Genecards database, and 322 overlapping targets were evident between these two sets (Figure 2A).

The PPI network, generated by STRING, contained 320 nodes and 6782 edges with an average node degree of 42. Core targets associated with many edges and nodes included TNF, IL6, and IL1β (Figure 2B). Details regarding the possible mechanisms through which CGE combats antioxidant stress and the active compounds present therein were obtained by constructing a compound–target network that included 416 potential hub targets together with 15 core compounds.

Of the identified compounds, mussaenoside exhibited the highest degree value, while GAPDH, TNF, AKT1, IL6, and IL1β were the targets that interacted with the most other targets and active compounds when focused on antioxidant stress. GO analyses demonstrated the enrichment of these genes in the response to drug, response to oxidative stress, response to lipopolysaccharide, cellular response to chemical stress, response to nutrient levels, and other biological processes (Figure 2C). They were also enriched in cellular component terms including membrane rafts, membrane microdomains, the membrane region, integral component of presynaptic membrane, and intrinsic component of presynaptic membrane. Finally, they were enriched in molecular functions that included nuclear receptor activity, ligand-activated transcription factor activity, and drug binding.

The KEGG analysis of the targets indicated enrichment in the AGE-RAGE axis in diabetes, as well as pathways associated with TNF, PI3K/AKT, and IL-17 signaling, prostate cancer, and lipids and atherosclerosis (Figure 2D). CGE thus exhibits anti-inflammatory activity linked to the regulation of the PI3K/AKT axis, although this hypothesis will require testing in further studies.

### 3.3. Molecular Docking

To confirm these network pharmacology results, molecular docking was used to assess interactions between AKT1 (PDB 4EJN) and the active compounds azafrin and mussaenoside to demonstrate affinity and detect the core binding sites. Azafrin and mussaenoside are the main active substances in the component targets, so they were selected for molecular docking. The results showed that azafrin presented with high affinity for AKT1 (Figure 2E and Table S1), with a binding energy of −6.35 kcal/mol. It was also able to bind to VAL (271) and LYS (158) through hydrogen bonding. Mussaenoside also bound to the LEU (295), LYS (297), CYS (310), ASP (274), LYS (20), GLU (17), and GLU (85) residues of AKT1 (Figure 2F and Table S1) with high affinity, with a binding energy of −5.02 kcal/mol, respectively. These findings suggest that core compounds including azafrin and mussaenoside may exhibit antioxidant activity by interacting with targets like AKT1.

### 3.4. Antioxidant Activity Analyses

**Figure 3:**
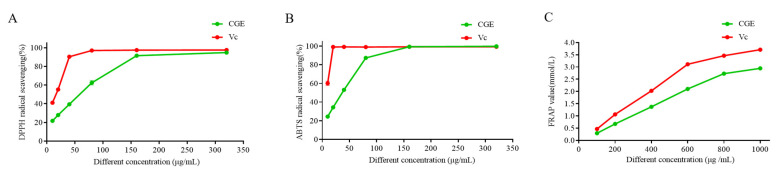
In vitro antioxidant assay analyses of CGE and Vc. DPPH (**A**), ABTS (**B**), and FRAP (**C**) free radical scavenging.

Iridoid glycosides derived from *C. grandiflora* Benth are closely linked to its ability to prevent or treat various chronic diseases. Indeed, iridoid glycosides exhibit an array of health benefits that can mitigate oxidative stress-associated disorders. Given the high iridoid glycoside levels therein, the samples prepared in this study thus hold great promise as antioxidant materials. As shown in Figure 3, various antioxidant assays, including ABTS, DPPH, and FRAP, were conducted for the CGE.

When testing the ability of the fractions to scavenge DPPH and ABTS, the rates for CGE and Vc rose dose-dependently (Figure 3A,B and Table S2). CGE exhibited the most robust radical scavenging activity relative with EC_50_ values of 72.922 ± 4.461 µg/mL (DPPH) and 47.447 ± 0.851 µg/mL (ABTS). The best free radical scavenging activity of CGE was evident at 320 μg/mL, achieving activity similar to that of V_C_. In line with the FRAP assay, CGE achieving a maximum FRAP value at 1 mg/mL (Figure 3C), in line with activity levels observed for Vc. CGE also presented with FRAP activity that was strongest in the 0.2–0.8 mg/mL range, with FRAP values rising from 0.671 to 2.729 mmol/L.

In light of these findings, CGE was chosen for further studies and potential utilization as a natural antioxidant.

### 3.5. CGE Protects Against Oxidative Injury in LPS-Treated RAW264.7 Cells

#### 3.5.1. Evaluation of the Effects of CGE on RAW264.7 Viability

**Figure 4:**
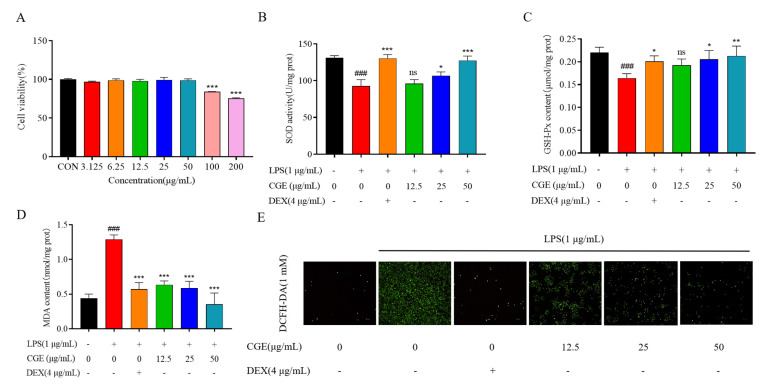
Impact of CGE on LPS-treated RAW264.7 cells. Cells were treated with 3.125–200 µg/mL CGE for 24 h. Cell viabilities were assessed by MTT assays (**A**). Following a 2 h treatment with a range of CGE doses (12.5–50 µg/mL), RAW264.7 cells were grown with LPS (1 µg/mL) for 18 h. Analyses of SOD (**B**), GSH-Px (**C**), and MDA levels (**D**). DCFH-DA staining was used to assess ROS biogenesis (**E**). Mean ± SD, *n* = 3. ###: *p* < 0.001 vs. control. *: *p* < 0.05, **: *p* < 0.01, ***: *p* < 0.001 vs. LPS. ns: no significance.

To ensure the safety of CGE before testing its bioactivity, an MTT assay was performed using RAW264.7 macrophages treated with CGE doses from 3.125 to 200 µg/mL (Figure 4A). These doses had no significant proliferation-inducing effect relative to control cells in the 3.125–6.25 µg/mL range, while significantly enhanced proliferation was evident in the 12.5–50 µg/mL range, and doses above 100 µg/mL significantly suppressed cellular proliferation (*p* < 0.05). All subsequent experiments were thus performed with CGE doses from 12.5–50 µg/mL.

#### 3.5.2. Analyses of LPS-Induced ROS Biogenesis

Oxidative stress is closely tied to many forms of disease, and efforts to prevent this stress are thus a key goal of functional food-based treatment [43]. LPS-treated RAW264.7 cells are commonly used to assess antioxidant activity in vitro. Here, the protective effects of CGE were thus evaluated using this model system (Figure 4). DCFH-DA was used to quantify ROS production as it only fluoresces when oxidized by ROS. As illustrated in Figure 4E, LPS markedly increased ROS levels as detected via laser scanning confocal microscopy relative to control cells, while CGE suppressed this ROS accumulation in a dose-dependent fashion.

#### 3.5.3. Analyses of MDA, SOD, and GSH-Px Concentrations in LPS-Stimulated RAW264.7 Cells

Free radicals include unpaired electrons, with the most common classes including hydroxy (OH^−^), peroxy (ROO.), and superoxide (O^2−^) radicals. After forming, these free radicals can enter and damage cellular structures in a manner counteracted by antioxidants [44]. MDA is a lipid peroxidation end product that develops in the context of free radicals or arachidonic acid, and serves as a robust biomarker of oxidative stress given that its levels rise in parallel with overall oxidative stress [45]. SOD is an enzyme that helps counteract oxidative stress, scavenging free radicals and converting superoxide radicals into hydrogen peroxide and oxygen [46]. Reduced glutathione (GSH) is responsible for removing reactive oxygen from cells [47,48]. To probe the antioxidant activity of CGE, the MDA, SOD, and GSH-Px contents of cells were analyzed to better understand how *C. grandiflora* Benth exerts its function. Commercial kits were used to quantify these levels in LPS and CGE-treated RAW264.7 macrophages, utilizing DEX as a positive control. As illustrated in Figure 4B–D, the activities of both SOD and GSH-Px were markedly decreased in response to LPS treatment whereas MDA levels rose over those in control cells (*p* < 0.05). CGE (12.5, 25, and 50 µg/mL) treatment, in contrast, enhanced SOD and GSH-Px activity while attenuating the MDA contents in these LPS-stimulated RAW264.7 cells.

CGE may thus be a key bioactive component of *C. grandiflora* Benth, contributing to its overall antioxidant activity through the scavenging of ROS. Overall, CGE was able to inhibit oxidative stress through the enhancement of SOD and GSH-Px activity together with reductions in MDA and ROS levels. CGE treatment may thus be a promising treatment for oxidative stress-related conditions that can improve human health and well-being.

### 3.6. CGE Exhibits Anti-Inflammatory Activity

#### 3.6.1. CGE Alters LPS-Induced NO and Inflammatory Mediator Production in RAW264.7 Cells

Excessive inflammation is linked to various diseases, such as cancer, inflammatory bowel disease, and obesity [49,50,51]. Drug-mediated treatment of inflammation has the potential to cause adverse side effects, spurring growing interest in herbal formulations. Many studies have demonstrated that plant-derived iridoid glycosides exhibit robust anti-inflammatory properties [52,53]. In this study, LPS-stimulated RAW264.7 macrophages were thus employed to evaluate CGE anti-inflammatory effects.

**Figure 5:**
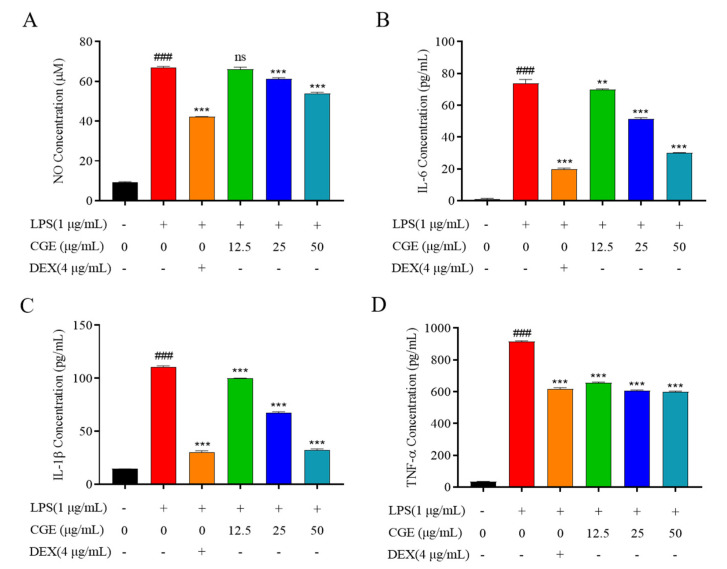
CGE exhibits anti-inflammatory activity. After pretreating RAW264.7 cells for 2 h with various CGE doses (12.5–50 µg/mL), they were grown with LPS (1 µg/mL) for 18 h. Supernatant NO levels were detected with Griess reagent (**A**). ELISAs were used to measure IL-6 (**B**), IL-1β (**C**), and TNF-α (**D**). Mean ± SD, *n* = 3. ###: *p* < 0.001 vs. control **: *p* < 0.01, ***: *p* < 0.001 vs. LPS. ns: no significance.

As a free radical that is transiently generated in most cell types, NO can function in many different biological contexts in response to LPS or other inflammatory stimuli such that it is regarded as a marker of inflammation [54,55]. NO measurement revealed that LPS exposure (1 µg/mL, 18 h) markedly raised NO biogenesis, while CGE treatment (12.5, 25, and 50 µg/mL) dose-dependently blocked the release of NO in these cells (Figure 5A), with DEX serving as a positive control.

Other key factors associated with inflammation such as cytokines can contribute to cellular and tissue damage through the induction of local tissue degeneration, proliferation, and exudate formation. Inhibiting these cytokines can aid in the effective treatment of many forms of inflammatory disease [37,56]. When the levels of these cytokines were analyzed in the established experimental system, LPS triggered the significant upregulation of TNF-α, IL-1β, and IL-6 in the cells following a 18 h treatment period, while CGE pretreatment dose-dependently inhibited the release of these three cytokines (Figure 5B–D). CGE thus appears to hold great promise as an efficacious anti-inflammatory agent.

#### 3.6.2. CGE Affects Inflammation-Associated Levels of iNOS and COX-2

**Figure 6:**
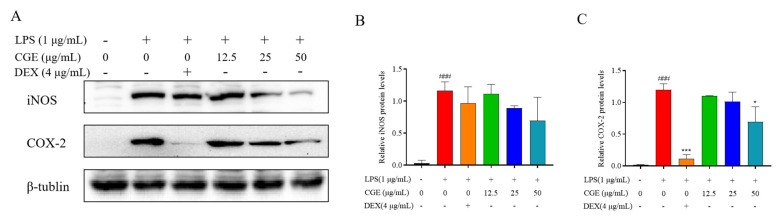
CGE modulates iNOS and COX-2 levels in LPS-stimulated RAW264.7 cells. After pretreating cells for 2 h with various CGE doses (12.5–50 µg/mL), they were treated for 18 h with 1 µg/mL LPS. iNOS and COX-2 proteins were detected by Western immunoblotting with corresponding quantification (**A**–**C**). Mean ± SD, *n* = 3. ###: *p* < 0.001 vs. control *: *p* < 0.05, ***: *p* < 0.001 vs. LPS.

Both COX-2 and iNOS serve as key inflammatory response regulators, with iNOS functioning by continuously producing NO, which in turn promotes COX-2 release, exacerbating ongoing inflammation [57,58]. Efforts to reduce iNOS and COX-2 activity are thus vital to achieve anti-inflammatory efficacy. To explore the degree to which decreased iNOS and COX-2 levels underlie the reduced NO production by CGE-treated cells, their protein levels were examined. CGE was ultimately found to reduce raised iNOS and COX-2 levels in the cells (Figure 6A–C), suggesting it can counteract their ability to promote vascular permeability and tissue damage.

#### 3.6.3. CGE Modulates the NF-κB Axis in RAW264.7 Cells

**Figure 7:**
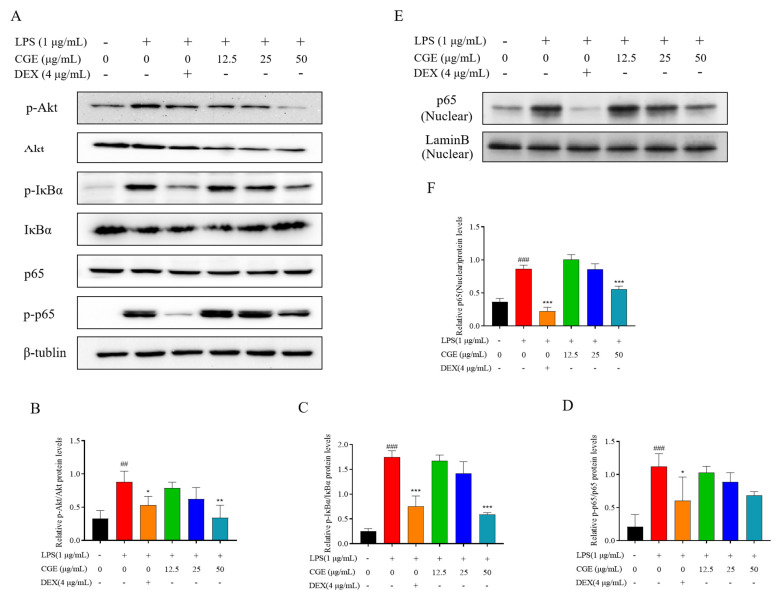
CGE influences NF-κB levels in LPS-treated RAW264.7 cells. After pretreating cells for 2 h with CGE (12.5–50 µg/mL), they were grown with 1 µg/mL LPS for 18 h. p-Akt, Akt, p65, p-p65, p-IκBα, IκBα, and LaminB levels were evaluated via Western immunoblotting with corresponding quantification (**A**–**F**). Mean ± SD, *n* = 3. ##: *p* < 0.01, ###: *p* < 0.001 vs. control. *: *p* < 0.05, **: *p* < 0.01, ***: *p* < 0.001 vs. LPS.

Network pharmacology established that Akt was an important CGE target linked to reductions in oxidative stress. To test this possible link, the concentrations of components of the PI3K-Akt axis were evaluated. Akt is also capable of influencing NF-κB signaling activity, which triggers inflammation and promotes inflammatory factor production [59]. To test this possibility, p-Akt, Akt, p65, p-p65 levels, and those of the key NF-κB suppressors IκBα and p-IκBα, were analyzed in these cells. Under normal conditions, NF-κB and IκBα are distributed in the cytoplasm, in a stable complex. When the cells are exposed to external stimuli, IκBα undergoes phosphorylation and P65 separates from IκBα and translocates to the nucleus, thus triggering inflammation. The results showed that treatment with LPS triggered a pronounced increase in IκBα phosphorylation and degradation, whereas CGE (12.5–50 μg/mL) suppressed these changes (Figure 7). LPS exposure significantly increased the phosphorylation of NF-κB p65 consistent with the enhancement of macrophage NF-κB, whereas these effects were blocked by CGE. Furthermore, we found that CGE was able to inhibit P65 transport from the cytoplasm to the nucleus in a dose-dependent manner (Figure 7E,F). Due to the known involvement of NF-κB in inflammation, this suggested that CGE is an inhibitor of its downstream genes due to its ability to suppress NF-κB, ultimately achieving anti-inflammatory efficacy.

#### 3.6.4. CGE Also Modulates Keap1-Nrf2 Pathway Signaling

**Figure 8:**
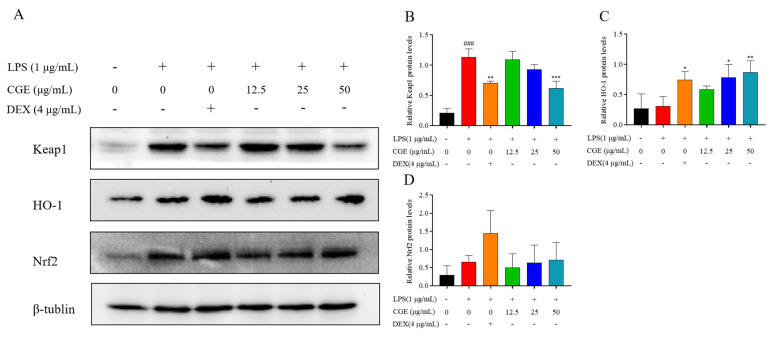
CGE modulates Keap1, HO-1, and Nrf2 levels in LPS-treated RAW264.7 cells. After pretreating cells for 2 h with various CGE doses (12.5–50 µg/mL), they were grown with 1 µg/mL LPS for 18 h. Keap1, HO-1, and Nrf2 levels were detected by Western immunoblotting with corresponding quantification (**A**–**D**). Mean ± SD, *n* = 3. ###: *p* < 0.001 vs. control *: *p* < 0.05, **: *p* < 0.01, ***: *p* < 0.001 vs. LPS.

In addition, signaling via Keap1-Nrf2 is a major mechanism through which cells counteract oxidative stress [60]. Accordingly, the levels of Keap1, Nrf2, and HO-1 were analyzed in these RAW264.7 cells. Normally, Nrf2 is bound to Keap1, releasing it only under conditions of oxidative stress whereupon it can induce the expression of HO-1 as well as other antioxidant genes, thereby helping to mitigate the source of this stress [61,62]. In these analyses, CGE increased Nrf2 expression and associated HO-1 levels while suppressing Keap1 expression (Figure 8A–D).

These analyses provided support for the ability of CGE to activate Keap1/Nrf2/HO-1 signaling, potentially thereby suppressing PI3K/AKT/NF-κB signaling such that CGE was able to mitigate oxidative stress and associated inflammatory factor production.

## 4. Conclusions

In summary, the structural features of the compounds present in plants and other foods strongly influence their bioactivity and associated applications. Here, 20 compounds were found to be present in CGE, and these compounds were predicted to positively affect oxidative stress and inflammation-related diseases through impacts on several signaling pathways, including the PI3K/AKT pathway. CGE also exhibited a high degree of nutritional value, serving as an important source of fiber, protein, minerals, and essential amino acids, which may contribute to its antioxidant activity and health benefits. It also presented with a good biosafety profile.

CGE also presented with a dose-dependent ability to scavenge free radicals and mitigate oxidative stress by reducing ROS. CGE was found to exert antioxidant effects through increases in SOD and GSH-Px activities together with the scavenging of MDA and ROS. CGE also reduced NO, TNF-α, IL-1β, and IL-6 expression, highlighting its robust anti-inflammatory properties. Mechanistically, CGE was able to mitigate oxidative stress and inflammation induced by LPS through reducing PI3K/AKT/NF-κB activity together with Nrf2/HO-1 activation. As possible bioactive compounds, azafrin and mussaenoside exhibited a high affinity for Akt1-related targets. These results provide clear scientific evidence that can support the further utilization of *C. grandiflora* Benth.

Together, these data demonstrate the antioxidant and anti-inflammatory properties of *C. grandiflora* Benth, offering a sound foundation for the further investigation of its use in health-promoting beverages, enriched foods, or dietary supplements.

## Figures and Tables

**Table 1:** Data corresponding to chemical compounds 1–20.

NO.	RT (Min)	Ionization Mode	Experimental *m/z*	MS/MS Fragments	Tentative Compound	Molecular Formula	Molecular Weight	Ref.
1	2.07	[M−H]^+^	146.9962	146,129,101,84	Glutamate	C_5_H_9_NO_4_	147	[33]
2	2.39	[M+H]^+^	183.0862	129,116,104,61	Mannitol	C_6_H_14_O_6_	182	[9]
3	5.69	[M+H]^+^	347.1256	347,311,268,168,61	Aucubin	C_15_H_22_O_9_	346	[4]
4	5.74	[M+K]^+^	389.1240	327,293,167,131,103	Vomilenine	C_21_H_22_N_2_O_3_	350	[34]
5	6.02	[M−H]^−^	577.1559	577,533,473,327,298	Vitexin 2″-O-rhamnoside	C_27_H_30_O_14_	578	[35]
6	7.15	[M−H]^−^	373.1134	373,248,198,149,105	Geniposidic acid	C_16_H_24_O_10_	374	[4]
7	7.86	[M−H]^−^	375.4306	375,220,162,84	Mussaenosidic acid	C_16_H_24_O_10_	376	[4]
8	9.95	[M+H]^+^	405.2121	405,287,225,207	Melasmoside	C_19_H_32_O_9_	404	[9]
9	10.21	[M+H]^+^	134.1559	134,116,105,88,70	Aspartic acid	C_4_H_7_NO_4_	133	[36]
10	10.95	[M+H]^+^	391.1619	391,373,229,193,179	Mussaenoside	C_17_H_26_O_10_	390	[4]
11	12.01	[M+Na]^+^	455.4532	455,413,121	2-methoxy-4-methy-lphenyl-O-*β*-D-apiofuranosyl-(1→6)-*β*-D-glucopyranoside	C_19_H_28_O_11_	432	[37]
12	12.65	[M+H]^+^	243.1581	225,127,189,165	Trihydroxy-β-violetone	C_13_H_22_O_4_	242	[38]
13	13.21	[M+CH_3_COO]^−^	669.1829	609,520,491,367,248	Rutin	C_27_H_30_O_16_	610	[39]
14	13.30	[M+H]^+^	132.1016	132,129,86	Leucine	C_5_H_9_NO_4_	131	[40]
15	15.70	[M+Na−H_2_O]^+^	437.2151	415,397,305,281,217	Centrantheroside A	C_21_H_36_O_9_	432	[9]
16	15.90	[M+CH_3_COO-H_2_O]^−^	401.1457	248,187,154	Catalpol	C_16_H_24_O_9_	360	[4]
17	21.46	[M+H]^+^	227.1641	227,209,188,141	Dihydroxy-*β*-violetone	C_13_H_22_O_3_	226	[38]
18	22.24	[M+CH_3_COO]^−^	491.1205	491,403,316,197	Sophoricoside	C_21_H_20_O_10_	432	[41]
19	26.21	[M−H]^−^	425.2707	425,384,355,311	Azafrin	C_27_H_38_O_4_	426	[5]
20	44.27	[M−H]^−^	455.3541	455,397,339,275	Ursolic acid	C_30_H_48_O_3_	456	[42]

**Table 2:** Data pertaining to chemical compounds 1–20.

Proximate Composition (%)		Minerals (mg per 100 g)		Amino Acids (mg per 100 g)	
Name	Content	Name	Content	Name	Content
Carbohydrates	43.00	Fe	292.72	Asp	3.79
Fiber	35.94	Mg	443.14	Thr	1.87
Ash	12.26	Na	61.74	Ser	2.93
Protein	8.77	P	151.89	Glu	4.08
Moisture	8.67	Ca	276.76	Gly	2.09
Lipids	0.04	K	760.02	Ala	1.51
		Cu	2.52	Val	1.74
		Zn	5.61	Met	1.01
		Cr	1.63	Ile	2.05
		Total	2601.03	Leu	3.19
				Tyr	2.18
				Phe	1.82
				His	0.14
				Lys	0.54
				Total	28.92

## Data Availability

Data are contained within the article and supplementary materials.

## Supplementary Materials

The following supporting information can be downloaded at: https://www.mdpi.com/article/10.3390/nu17050925/s1, Table S1: Molecular docking of Azafrin and Mussaenoside with AKT; Table S2: EC50 values for CGE and VC (DPPH and ABTS).
