# Supplementary material for: Evaluating the Components, Nutrients, and Antioxidant and Anti-Inflammatory Properties of Centranthera grandiflora Benth Extracts
Source: Nutrients. 2025 Mar 6;17(5):925. doi: 10.3390/nu17050925 (PMC11901887; doi:10.3390/nu17050925)
Supplement: Supplementary file 1 [file nutrients-17-00925-s001.zip › nutrients-3461501-supplementary.pdf]

Supplementary documents

The nutritional value, chemical composition, the antioxidant and anti-inflammatory activities of *Centranthera grandiflora* Benth extracts

Supporting information

Table S1. Molecular docking of Azafrin and Mussaenoside with AKT.

Name	Affinity (kcal/mol))	Libdock score
Mussaenoside	-5.02	-130.724
Azafrin	-6.35	-132.957

Table S2. EC_{50} values for CGE and V_C (DPPH and ABTS).

Sample	DPPH (EC_{50}, μg/mL)	ABTS (EC_{50}, μg/mL)
CGE	72.922	47.447
V_C	13.622	5.1
